# Resistome and Virulome of Multi-Drug Resistant *E. coli* ST131 Isolated from Residents of Long-Term Care Facilities in the Northern Italian Region

**DOI:** 10.3390/diagnostics12010213

**Published:** 2022-01-16

**Authors:** Sabrina Cherubini, Mariagrazia Perilli, Anna Maria Azzini, Evelina Tacconelli, Laura Maccacaro, Alda Bazaj, Laura Naso, Gianfranco Amicosante, LTCF-Veneto Working Group, Giuliana Lo Cascio, Alessandra Piccirilli

**Affiliations:** 1 Department of Biotechnological and Applied Clinical Sciences, University of L’Aquila, 67100 L’Aquila, Italy; sabrina.cherubini@graduate.univaq.it (S.C.); mariagrazia.perilli@univaq.it (M.P.); gianfranco.amicosante@univaq.it (G.A.); 2 Department of Diagnostic and Public Health, Infectious Disease Section, University of Verona, 37134 Verona, Italy; annamaria.azzini@univr.it (A.M.A.); evelina.tacconelli@univr.it (E.T.); 3 Microbiology and Virology Unit, Department of Pathology and Diagnostic, Azienda Ospedaliera Universitaria Integrata di Verona, 37134 Verona, Italy; laura.maccacaro@aovr.veneto.it (L.M.); alda.bazaj@univr.it (A.B.); laura.naso@hotmail.it (L.N.); g.locascio@ausl.pc.it (G.L.C.); 4 Microbiology and Virology Unit, AUSL Piacenza, 29121 Piacenza, Italy

**Keywords:** *E. coli*, WGS, antibiotic resistance genes, β-lactamases, virulome

## Abstract

Long-term care facilities (LTCFs) are important reservoirs of antimicrobial-resistant (AMR) bacteria which colonize patients transferred from the hospital, or they may emerge in the facility as a result of mutation or gene transfer. In the present study, we characterized, from a molecular point of view, 43 *E. coli* strains collected from residents of LTCFs in Northern Italy. The most common lineage found was ST131, followed by sporadic presence of ST12, ST69, ST48, ST95, ST410 and ST1193. All strains were incubators of several virulence factors, with *iss*, *sat*, *iha* and *senB* being found in 84%, 72%, 63% and 51% of *E. coli*, respectively. Thirty of the ST131 analyzed were of the O25b:H4 serotype and H30 subclone. The ST131 isolates were found to be mainly associated with IncF plasmids, CTX-M-1, CTX-M-3, CTX-M-15, CTX-M-27 and *gyrA/parC/parE* mutations. Metallo-β-lactamases were not found in ST131, whereas KPC-3 carbapenemase was found only in two ST131 and one ST1193. In conclusion, we confirmed the spread of extended-spectrum β-lactamase genes in *E. coli* ST131 isolated from colonized residents living inside LTCFs. The ST131 represents an incubator of fluoroquinolones, aminoglycosides and other antibiotic resistance genes in addition to different virulence factors.

## 1. Introduction

In the European Union/European Economic Area (EU/EEA) prior to December 2019, there were an estimated 2.9 million residents in 43,000 LTCFs, representing approximately 0.7% of the total population [1]. Reports from 2019 estimate that around 5.6% of the Italian population was potentially dependent, and 28.7% of them aged more than 65 in need of long-term care facilities (LTCFs) [1]. LTCFs are considered important reservoirs of antimicrobial-resistant (AMR) bacteria because of their colonization of residents discharged from the hospital, or they become colonized inside the facility as a result of antibiotic selective pressure [2,3,4]. The EU Centre for Disease Prevention and Control (ECDC) supports point prevalence surveys of healthcare-associated infections (HAIs) and antimicrobial use in European LTCFs [5]. This program monitors the burden of HAIs and antimicrobial resistance inside LTCFs, comparing data from different countries and aiding in the implementation of multimodal interventions to reduce them. Infections of the urinary tract, lower respiratory tract and gastroenteric tract caused by *Enterobacterales*, in particular *Klebsiella pneumoniae* and *Escherichia coli*, are the most frequent [6,7]; in particular, extra-intestinal pathogenic *E. coli*, resistant to numerous classes of antibiotics, is the leading cause of serious infections among LTCF residents, and the recent emergence and spread of extended-spectrum β-lactamases (ESBLs) and/or carbapenemase-producing *Enterobacterales* firmly reduced the therapeutic options. The aim of the present study was to characterize the resistome and virulome of *E. coli* strains isolated from residents of LTCFs in Veneto, a Northern Italian region. The study was assessed using a next-generation sequencing platform.

## 2. Materials and Methods

### 2.1. Setting

Between July 2018 and June 2019 we conducted a point prevalence survey to assess both the frequency of healthcare-related infections and the enteric colonization status by multi-antibiotic-resistant (MDR) Gram negative bacteria in the population of elderly residents of 27 long-term care facilities (LTCF) in the Veneto Region. Joining the survey was voluntary, but at least one facility for each province of the region was enrolled. The 27 facilities were not involved simultaneously but at different times based on the local Ethic Committee’s approval, the willingness of local personnel to collaborate with researchers in the collection of the study-specific biological samples and the possibility of the reference microbiology laboratory to accept and process them. The survey was proposed to all patients who were hospitalized for at least 48 h and physically present in the facility at 8:00 a.m. of the survey day. Only patients who were able to provide written informed consent, or whose legal guardian consented, were enrolled. The survey was carried out in a single day for each facility. In the case of facilities with a high number of residents, the survey lasted several consecutive days, with the obligation to complete the evaluation of all subjects in each ward in a single day. For each enrolled patient, 118 variables were collected, including the type and etiology of concurrent infections and respective antibiotic therapy, any antibiotic treatments in the previous 3 months, hospital admissions and surgery in the previous 12 months and invasive medical devices in situ. For each enrolled host, a rectal swab was performed to assess the status of colonization by Gram negative MDR (*Enterobacterales* and non-fermenter Gram negatives producing ESBL and/or resistant to carbapenems).

### 2.2. Strains Selection and Antimicrobial Susceptibility

The strain selection was performed by inoculating the rectal swabs onto ChromID ESBL agar (bioMérieux, Marcy l’Etoile, France) and on Mac Conkey agar. Identification of the isolates was carried out in an automated Vitek2 System (bioMérieux, Marcy l’Etoile, France). Antimicrobial susceptibilities were performed on a Vitek2 system (version 9.02, bioMérieux, Marcy lʹEtoile, France). The strains that showed resistance to carbapenems were also analyzed with an immunochromatographic lateral flow assay Carba5 (NG Biotech, Guipry, France). Resistance to antibiotics was interpreted according to the EUCAST criteria. The *E. coli* that showed susceptibility to third-generation cephalosporins and carbapenems were excluded from the study.

### 2.3. DNA Extraction and Whole Genome Sequencing (WGS)

Total genomic DNA was extracted from 1 mL of an overnight bacterial culture using a MagMAX Microbiome Ultra Nucleic Acid Isolation kit (Applied Biosystems and ThermoFisher Scientific, Monza, Italy) following the manufacturer’s instructions. Whole genome sequencing (WGS) was performed using the Illumina Miseq platform with a 2 × 300 paired-end run.

### 2.4. Bioinformatics Analysis

Quality control and sequences filtering were checked using DRAGEN FastQC + MultiQC v3.6.3 (https://basespace.illumina.com/apps/10562553/DRAGEN-FastQC-MultiQC, access date: 9 September 2021) and assembled with Velvet v1.2.10 (https://basespace.illumina.com/apps/8556549/Velvet-de-novo-Assembly, access date: 9 September 2021) [8]. Acquired antimicrobial resistance genes and chromosomal point mutations (*gyrA, parC* and *parE* genes) were identified using ResFinder 4.1 (https://cge.cbs.dtu.dk/services/ResFinder/, access date: 13 September 2021) [9] and virulence genes using VirulenceFinder 2.0 (https://cge.cbs.dtu.dk/services/VirulenceFinder/, access date: 13 September 2021). Serotypes and fimH types were determined using SerotypeFinder 2.0 and FimTyper 1.0 (http://genomicepidemiology.org/services/, access date: 14 September 2021) tools, respectively. Multilocus sequence typing (MLST) was performed using a BLAST-based approach [10]. The allele number of the seven housekeeping genes, *adk*, *fumC*, *gyrB*, *icd*, *mdh*, *purA* and *recA*, and the Sequence Type (ST) of each isolate was determined by combining seven allelic profiles in MLST *E. coli* Atchman database (https://pubmlst.org/bigsdb?db=pubmlst_mlst_sqdef&page=schemeInfo&scheme_id=4, access date: 20 September 2021). PlasmidFinder 2.1 was used to detect the incompatibility groups of plasmids. Identified plasmids of the IncF, IncH1, IncH2, IncI1, IncN or IncA/C type were subtyped by pMLST 2.0 (https://cge.cbs.dtu.dk/services/pMLST/, access date: 21 September 2021) [11].

## 3. Results

### 3.1. Strains Selection and Antimicrobial Susceptibility

Using a selective medium, 831 *E. coli* strains were isolated from 1981 residents of 27 LTCFs, and 43 strains collected from 13 LTCFs were randomly selected for WGS analysis. Overall, 100% of them were resistant to oxyimino-cephalosporins (cefotaxime and ceftazidime), 72% to ciprofloxacin and 21% to carbapenems (meropenem and/or imipenem), and 46% were also resistant to piperacillin-tazobactam association.

### 3.2. WGS, MLST and Serotype

*E. coli* strains were analyzed by whole-genome sequencing, which provides information about the sequence typing (MLST) of *E. coli*, serotypes, virulence genes, plasmid replicons, pMLST and antibiotic resistant genes (ARGs). The whole-genome size of the 43 *E. coli* ranged from 4.9 to 10 Mb. On the basis of the Achtman scheme, which considers *adk*, *fumC*, *gyrB*, *icd*, *mdh*, *purA* and *recA* housekeeping genes, the predominant ST found was ST131 (74% of isolates) followed by ST12 (5% isolates), ST69 (7% of isolates), ST48 (2% isolates), ST95 (2% isolates), ST410 (2% isolates) and ST1193 (7% isolates) (Table 1). The serotype of each *E. coli* was also determined. Thirty ST131 isolates showed a O25b:H4 serotype with the fimbria variant fimH30 and two O61:H4 serotypes with fimH94. The ST12 belonged to the O4:H5 serotype with fimH5 and fimH240, the ST48 belonged to the O137:H4 with fimH54, the ST69 belonged to O15:H18 with fimH27 and O44-O77:H18 with fimH27, the ST95 belonged to O18:H7 with fimH15, the ST410 belonged to H21 with fimH24 and the ST1193 belonged to O75:H5 with fimH64.

### 3.3. Virulence Genes

The virulence genes found in the *E. coli* isolates analyzed in this study were the following: *sat* (secreted autotransporter toxin), *iha* (adhesion-siderophore receptor), *iss* (increased serum survival), *senB* (plasmid encoded enterotoxin), *gad* (glutamate decarboxylase), *astA* (east-1 heat-stable toxin), *lpfA* (long polar fimbriae), *air* (enteroaggregative immunoglobulin repeat protein), *eilA* (Salmonella HilA homolog), *cnf1* (cytotoxic necrotizing factor), *vat* (vacuolating autotransporter toxin), *ireA* (iron-regulated outer membrane virulence protein), *iroN* (salmchelin siderophore receptor), *sfaS* (S fimbriae), *nfaE* (non fimbrial adhesion) and *mcmA*, *mchB*, *mchC* and *mchF*, which are the microcin H47 system virulence genes (Table 2). The increased serum survival (*iss*), secreted autotransporter toxin (*sat*), adhesion-siderophore receptor (*iha*) and plasmid encoded enterotoxin (*senB*) were found in 84%, 72%, 63% and 51% of *E. coli*, respectively. As shown in Table 1, in the *E. coli* ST12, ST69 and ST95, different virulence genes were found; on the contrary, only two virulence genes were identified in ST410 and ST1193.

### 3.4. Plasmid Replicons and pMLST

The different incompatibility plasmids IncI1, IncFII(29), IncFII(pRSB107), IncFIB, IncFIA, IncN, IncF, IncB/O/K/Z, IncQ1, IncX1, IncX3, IncX4, Col(MG828), Col156, Col8282 and ColRNAI were detected in all *E. coli* analyzed (Table 3). Each *E. coli* isolate harbored more than one type of plasmid. Overall, IncFII was the predominant plasmid, found in 84% of *E. coli*, followed by Col (Col(MG828), Col156, Col8282) in 76%, IncFIA in 64%, IncFIB in 60%, ColRNAI in 40%, IncN in 28%, IncI1 in 24%, IncX (IncX4, IncX3 and IncX1) in 24% and IncB/O/K/Z in 12% of *E. coli*. The pMLST showed that IncF was represented by several lineages such as fii_2, fii_24, fii_29, fia_1, fib_1, fib_10, F1:A2:B20, F1:A20:B31, F29:B10, F31:A20:B1, F1:A2, F4:A19, F2:A1, F1:A1:B10 and F46:A6:B47, whereas IncN plasmid was represented by the ST7 lineage. In detail, the pMLST of ST131 isolates revealed the presence of F1:A2:B20 (ten isolates), F1:A20:B31 (three isolates), F29:B10 (one isolate), F31:A20:B1 (one isolate), F1:A2 (one isolate), F2:A1 (three isolates) and F1:A1:B10 (two isolates) (Table 3).

### 3.5. Antibiotic Resistance Genes (ARGs)

The aminoglycoside resistance genes *aadA1*, *aadA2*, *aadA5*, *aacA1*, *aacA4*, *aac(3)-IId*, *aph(3′)-XV*, *aph(3′)-Ia* and *aph(3′)-IIa* and the bi-functional gene *aac(6′)Ib-cr* were found in all *E. coli* analyzed (Table 3). Fluoroquinolone resistance was plasmid-mediated by the presence of *qnr* elements. In particular, the *qnrS1* was identified in ten *E. coli*, whereas *qnrB19* and *qnrB66* were found in two and one isolates, respectively. In one isolate of *E. coli*, the simultaneous presence of *qnrS1* and *qnrB19* was identified. The most common mechanism of sulfamethoxazole/trimethoprim resistance was represented by the acquisition of dihydrofolate reductase *dfrA1*(1 isolate), *dfrA14* (8 isolates) and *dfrA17* (19 isolates). Other antibiotic resistance genes were the following: *mphA* (macrolide resistance) detected in 23 *E. coli*, *sul1*/*sul2* (suphonamide resistance) detected in 26 isolates, *catB2*/*catB4* (chloramphenicol resistance) detected in 17 isolates, *strA*/*strB* (streptomycin resistance) detected in 17 isolates and *tet*(*A*)/*tet*(*B*) (tetracycline resistance) detected in 22 isolates. The lincosamide nucleotidyltransferase gene (*lnu*(*F*)) was found in one ST48 isolate. All *E. coli* strains produced one or more β-lactamases belonging to molecular classes A, B, C and D. Consistent with the results in this study, the ESBLs were the most widespread, and they included enzymes of the CTX-M-1 group (CTX-M-1, CTX-M-3 and CTX-M-15) in 21 isolates, CTX-M-27 (belonging to CTX-M-9 group) in 12 isolates and SHV-12 in 3 isolates (Table 4). The CTX-M enzymes were found only in ST131 strains. Overall, 37 out 43 *E. coli* (87%) were positive to ESBLs. The VIM-1 enzyme was found in three ST69, one ST12 and one ST95. The *E. coli* ST410 produced the metallo-β-lactamase NDM-4 and CMY-42 class C enzyme. The KPC-3 carbapenemase was identified in three isolates, specifically two ST131 and one ST1193. The OXA-1 and OXA-9 were found in 12 and 3 isolates, respectively, and in association with other β-lactamase genes (Table 4).

### 3.6. Chromosomal Fluoroquinolones Resistance

Resistance to fluoroquinolones was also mediated by mutations in *gyrA*, *parC* and *parE.* As shown in Table 3, the S83L/D87N in *gyrA*, S80I/E84V in *parC* and I529L in *parE* were the most common substitutions found in twenty-nine ST131. The S83L/D87N in *gyrA*, S80I in *parC* and L416F in *parE* mutations were found in three ST1193. The S83L/D87N in *gyrA*, S80I in *parC* and S458A in *parE* mutations were identified in one ST410. Two isolates of *E. coli* ST69 showed the S83L and D87N mutations in *gyrA* and S80I in *parC*.

## 4. Discussion

Herein, we used NGS-technology to investigate the resistome, virulome and genetic diversity of multi-drug resistant *E. coli* collected from 13 LTCFs in the Veneto region. The majority of *E. coli* analyzed belongs to the ST131 lineage. From a clinical point of view, ST131 is an extra-intestinal pathogenic bacterium which lives in the digestive tract, but it could also mediate its pathogenicity in the urinary tract and in the blood stream [12]. Almost all ST131 (30 out of 32) analyzed were of the O25b:H4 serotype and H30 subclone. The fimH allele, encoding for the virulence factor used by bacteria to attach the host tissue, has been used to phylogenetically classify the ST131 isolates in three clades (A, B, C), being also based on antibiotic resistance genes [13,14]. As reported by Pitout et al., ST131 clades C1 and C2 are fluoroquinolone resistant, and C2 has a strong association with CTX-M production [14]. Several studies have proven that the most prevalent subclonal lineage of *E. coli* ST131 is fimH30, which is also associated with a specific mutation in *gyrA* and *parC*, conferring chromosomal resistance to fluoroquinolones [15,16]. Some *E. coli* lineages were identified only in one LTCF; this is the case for ST12, ST69 and ST95, found only in SSL_BL, the ST48 in SAF_VE and ST410 in POCS_VR. The ST410 lineage seems to be phylogenetically older than ST131, but because of its virulence profile, ST131 is globally distributed in several environments over the world [17]. The feature of ST410 strains, in contrast to ST131, is the resistance to carbapenems [17]. In the present study, the ST410-H24 strain showed resistance to carbapenems, producing NDM-4 metallo-β-lactamase, and to fluoroquinolones via plasmid *qnrS1* and to chromosomes by mutations in *gyrA*, *parC* and *parE*. The ST131 isolates were found to be associated with IncF plasmids, ESBLs (CTX-M-1-group, CTX-M-27) and chromosomal resistance to fluoroquinolone (*gyrA/parC/parE* mutations) [18]. They also possessed *aac(6′)-Ib-cr*, *catB4*, OXA-1/OXA-9 and other ARGs which reduced susceptibility to aminoglycoside, chloramphenicol, oxacillin and other classes of antibiotics. In ST131, we found the predominance of CTX-M variants (94% of isolates) followed by OXA-1/OXA-9 (47% of isolates). As previously reported, CTX-M-15 and CTX-M-27 alleles are associated with the ST131 clade C2 and ST131 clade C1 [19,20,21]. The CTX-M-27 is predominantly associated with the F1:A2:B20 replicon, as reported in other ST131 strains isolated worldwide [22]. The CTX-M-27 belongs to subgroup CTX-M-9, and it showed two amino acid substitutions from CTX-M-9 (A231V and D240G) and one from CTX-M-14 (D240G). CTX-M-27 was found, not only in clinical strains, but also in bacteria isolated from food-producing animals, livestock and environment [23,24,25]. Metallo-β-lactamases were not found in ST131, and this is in agreement with results reported in different countries [13]. Similarly, the KPC-3 carbapenemase was found only in three *E. coli* strains (ST131 and ST1193) collected in CDS_RO, IPABRT_VI and PCS_VR LTCFs. In a previous paper where we characterized the resistome of *K. pneumoniae* isolates, we found, in the same LTCFs, KPC-3 producing *K. pneumoniae* [26]. The *E. coli* and *K. pneumoniae* strains harbored the same IncFIB(pQil) plasmid. We speculate that, in the same LTCF, a transfer of the *bla*_KPC-3_ gene, presumably located in IncFIB(pQil) plasmid, may have occurred between *E. coli* and *K. pneumoniae*.

## 5. Conclusions

In this study, we confirmed the spread of extended-spectrum β-lactamases and carbapenemases in *E. coli* isolated from colonized residents living inside LTCFs. In particular, the CTX-M-enzymes belonging to sub-groups 1 and 9 represent the ESBLs mainly found in our strains. In the past decade, the CTX-M-enzymes have been increasingly detected in *E. coli* worldwide. A low percentage of carbapenemases, including KPC-, VIM- and NDM-variants, was identified in *E. coli* collected in the LTCFs included in this survey. The ST131 represents an incubator of fluoroquinolones, aminoglycosides and other antibiotic resistance genes and, in addition, different virulence factors. ST131 is composed by five clades (A, B, C0, C1 and C2), and clade C seems to be more fit than the others. The clade C showed resistance to fluoroquinolones and was able to acquire IncF plasmids, giving to *E. coli* a rapid and continual adaptation to different environments. Emerging evidence worldwide suggests that LTCFs are important reservoirs for ARGs’ dissemination. The presence of antimicrobial-resistant pathogens in LTCFs has serious consequences not only for residents but also for the LTCF as an organization, both in terms of internal strategies to contain and possibly reduce these pathogens and the acceptance of colonized patients from the hospital.

## Figures and Tables

**Table 1:** Genome analysis of *E. coli* isolated from residents of 13 LTCFs (Northern Italian Region).

LTCFs Code	No. Isolates	Genome Size (bp)	MLST *	Serotype	fimH	Virluence Genes
SSL_BL	2	5.360.264	ST69	O15:H18	27	*gad, air, lpfA, eilA, iss, iha, sat, senB*
	1	5.064.257	ST131	O25b:H4	30	*sat, iss, iha, gad*
	3	5.318.811	ST131	O25b:H4	30	*sat, iss, cnf1, senB*
	1	5.428.542	ST69	O17/O44-O77:H18	27	*eilA, gad, lpfA, sat, air, senB, iss, iha*
	1	5.506.897	ST131	O25b:H4	30	*senB, iss, sat, iha, cnf1*
	1	5.102.820	ST131	O25b:H4	30	*sat, iss, iha, gad*
	1	5.257.199	ST95	O18:H7	15	*mchC, senB, vat, ireA, iroN, iss, sfaS, gad, mchF*
	1	5.278.074	ST12	O4:H5	204	*cnf1, iroN, mcmA, mchF, mchC, mchB, vat, iss, gad*
	1	5.110.437	ST12	O4:H5	5	*cnf1, iroN, mcmA, mchF, mchC, mchB, vat, ireA*
ISRAA_TV	1	5.117.808	ST131	O25b:H4	30	*senB, iss, sat, iha*
SAF_VE	1	5.003.689	ST48	O137:H4	54	*mchF, gad, cma, iss, iroN*
CRMC_VE	1	5.143.042	ST131	O25b:H4	30	*iha, senB, sat, iss*
	1	5.126.057	ST131	O25b:H4	30	*sat, gad, iha, senB, iss*
CDS_RO	2	10.087.318	ST131	O61:H4	94	*astA, iss, iha, sat*
	1	5.158.809	ST1193	O75:H5	64	*sat, iha*
	1	5.151.322	ST131	O25b:H4	30	*iha, sat, iss*
IPABMC_VI	1	5.362.937	ST131	O25b:H4	30	*sat, senB, astA, iha, iss*
	2	5.045.886	ST131	O25b:H4	30	*astA, gad, iha, senB, iss*
	1	5.277.401	ST131	O25b:H4	30	*senB, iha, sat, astA, iss*
IPABRS_VI	2	5.238.706	ST131	O25b:H4	30	*gad, iha, sat, astA, iss*
	2	5.131.308	ST1193	O75:H5	64	*vat*
IPABRT_VI	2	4.999.937	ST131	O25b:H4	30	*sat, iss, senB*
	2	5.120.568	ST131	O25b:H4	30	*sat, iss, senB, iha, gad*
	1	4.865.293	ST131	O25b:H4	30	*iss, senB, iha*
	1	5.080.670	ST131	O25b:H4	30	*iss, cnf1*
IPABSC_VI	2	5.165.353	ST131	O25b:H4	30	*sat, iss, senB, iha*
	1	5.052.997	ST131	O25b:H4	30	*sat, iss, senB, iha*
POBB_VR	1	5.254.036	ST131	O25b:H4	30	*sat, iss, senB, iha, nfaE*
POVI_VR	1	5.353.306	ST131	O25b:H4	30	*iss, iha, sat, cnf1*
POSC_VR	2	3.813.125	ST131	O25b:H4	30	*gad, iss, cnf1, iha*
POCS_VR	1	4.907.147	ST410	H21	24	*gad, lpfA*
	1	5.230.571	ST131	O25b:H4	30	*sat, iha, iss*

For legal reasons, we used only the acronym of the 12 LTCFs. * MLST: Multilocus sequence typing.

**Table 2:** Distribution of virulence genes in different *E. coli.*

Virulence Gene	Target Class	Total No. 43/(%)
*air*	Enteroaggregative immunoglobulin repeat protein	2 (5%)
*astA*	East-1 heat stable toxin	7 (16%)
*cma*	Colicin M	2 (5%)
*cnf1*	Cytotoxic necrotizing factor	9 (21%)
*eilA*	Salmonella HilA homolog	3 (7%)
*gad*	Glutamate decarboxylase	10 (23%)
*iha*	Adhesion-siderophore receptor	27 (63%)
*ireA*	Iron-regulated outer membrane virulence protein	3 (7%)
*iroN*	Salmochelin siderophore receptor	7 (16%)
*iss*	Increased serum survival	36 (84%)
*lpfA*	Long polar fimbriae	5 (12%)
*mchB*	Microcin H47 system	3 (7%)
*mchC*	Microcin H47 system	5 (12%)
*mchF*	Microcin H47 system	7 (16%)
*mcmA*	Microcin H47 system	3 (7%)
*nfaE*	non-fimbrial adhesion	2 (5%)
*sat*	Secreted autotransporter toxin	31 (72%)
*senB*	Plasmid encoded enterotoxin	22 (51%)
*sfaS*	S fimbriae	2 (5%)
*vat*	Vacuolating autotransporter toxin	7 (16%)

**Table 3:** Resistome pattern of *E. coli* isolates from LTCFs in Northern Italian region.

LTCFs Code	No. Isolates	Plasmid Replicons/pMLST	Β-Lactam Resistant Genes	Other Antibiotics Resistant Genes	Chromosomal Point Mutation
SSL_BL	2	IncX4, IncI1, IncFII(29), IncN, IncFIB(AP001918), Col156 /IncF: fii_29, fib_10 IncN: ST7	*bla_VIM-1_*	*aacA4, aadA1, mph(A), sul1, dfrA14, qnrS1, aac(6′)Ib-cr, catB2*	*gyrA* D87N *parC* S80I
	1	Col(MG828),Col156, IncFII(pRSB107), IncFIA, IncN /IncF: F1:A2:B20	*bla_CTX-M-3_*	*aadA5, sul1, dfrA17*	*gyrA* S83L/D87N *parC* S80I/E84V *parE* I529L
	3	IncFII, Col156, IncFIB(AP001918), IncFIA /F1:A20:B31	*bla_CTX-M-15_, bla_OXA-1_*	*aac(6′)Ib-cr, aadA5, aac(3)-IIa, mph(A), sul1, dfrA17, tet(A), catB4*	*gyrA* S83L/D87N *parC* S80I/E84V *parE* I529L
	1	Col156, Col8282, IncI1, ColRNAI, IncB/O/K/Z, IncFIB(AP001918), IncFII(29), IncN /IncF: F29:B10 IncI1: arda_4, pill_6, repi1_1, sogs_3, trba_6 IncN: ST7	*bla_VIM-1_, bla_TEM-1B_*	*aph(3′)-XV, aac(6′)Ib-cr, strA, strB, aacA4, aadA5, aadA1, mph(A), sul1, sul2, dfrA17, qnrS1, catB2*	*gyrA* S83L
	1	IncFII, Col156, IncB/O/K/Z, IncFIB(AP001918), IncFIA /F31:A20:B1	*bla_CTX-M-15_, bla_OXA-1_*	*aac(6′)Ib-cr, aac(3)-IIa, catB4*	*gyrA* S83L/D87N *parC* S80I/E84V *parE* I529L
	1	IncFII(pRSB107), Col(MG828), ColRNAI, IncFIA /F1:A2	*bla_CTX-M-27_*	*strA, strB, sul2, tet(A), aadA5, mph(A), sul1, dfrA17*	*gyrA* S83L/D87N *parC* S80I/E84V *parE* I529L
	1	ColRNAI, IncFII(pCoo), IncN, Col156 /IncF: F29:B10 IncN: ST7	*bla_VIM-1_*	*aac(6′)Ib-cr, aacA4, aacA1, mph(A), sul1, dfrA14, qnrS1, catB2*	None
	1	IncFIB(AP001918), IncI1, IncQ1, InFII	*bla_TEM-1B_, bla_CTX-M-1_*	*aadA1, strA, strB, sul1, sul2, dfrA1, qnrS1, tet(A)*	None
	1	IncFIA(HI1), IncFII, IncN /IncF: F4:A19 IncN: ST7	*bla_VIM-1_*	*aac(6′)Ib-cr, aadA1, aph(3′)-XV, aacA4, mph(A), sul1, dfrA14, qnrS1, catB2*	None
ISRAA_TV	1	IncFII (pRSB107), IncB/O/K/Z, IncFIA, IncFIB (AP001918), Col(MG828), Col156 /F1:A2:B20	*bla_CTX-M-27_*	*strA, strB, sul2, tet(A)*	*gyrA* S83L/D87N *parC* S80I/E84V *parE* I529L
SAF_VE	1	IncFII, IncI1, IncX1, IncFIB(AP001918) /IncF: fib_1, fii_24	*bla_SHV-12_, bla_TEM-1B_*	*strA, strB, aadA2,* *aph(3′)-Ia, lnu(F), qnrB19, qnrS1, tet(B)*	None
CRMC_VE	1	IncFII(pRSB107), Col156, IncX4, IncFIA, IncFIB (AP001918), Col(MG828) /F1:A2:B20	*bla_CTX-M-27_*		*gyrA* S83L/D87N *parC* S80I/E84V *parE* I529L
	1	IncFII(pRSB107),Col156, IncX4, IncFIA, IncFIB (AP001918) /F1:A2:B20	*bla_CTX-M-27_*		*gyrA* S83L/D87N *parC* S80I/E84V *parE* I529L
CDS_RO	2	Col(MG828), IncX4, IncI1, Col8282,IncFIA,IncFII(pRSB107), IncN, ColRNAI, Col(KPHS6), p0111, Col156 /IncF: F2:A1 IncI1: ST57	*bla_TEM-1B_, bla_CTX-M-3_, bla_OXA-1_*	*aph(3′)-IIa, aac(6′)-Ib-cr, catB4*	None
	1	Col156, Col(MG828), IncFIA, IncFII /F2:A1	*bla_CTX-M-15_, bla_OXA-1_*	*aac(6′)-Ib-cr, catB4*	*gyrA* S83L/D87N *parC* S80I/E84V *parE* I529L
	1	IncFIB(pQil),Col(BS512), IncFIA, IncFIB(AP001918) /IncF: A1:B10	*bla_TEM-1A_, bla_KPC-3_, bla_OXA-9_*	*aac(3)-IId*	*gyrA* S83L/D87N *parC* S80I *parE* L416F
IPABMC_VI	1	Col(MG828), IncX4, IncFII(29), Col156, ColRNAI, IncFIB(AP001918) /F29:B10	*bla_TEM-1B_, bla_CTX-M-15_*	*aac(3)-Id, strA, strB, aadA5, mph(A), sul1, sul2, dfrA17, tet(A)*	*gyrA* S83L/D87N *parC* S80I/E84V *parE* I529L
	2	IncFII(29), IncFIB(AP001918), Col8282, ColRNAI, Col156 /F29:B10	*bla_CTX-M-15_,* *bla_TEM-1B_*	*strA, aac(3)-IId, strB, aadA5, mph(A), sul2, sul1, dfrA17,tet(A)*	*gyrA* S83L/D87N *parC* S80I/E84V *parE* I529L
	1	IncFII(29), IncFIB(AP001918), Col156, ColRNAI, Col8282 /IncF: F29:B10	*bla_TEM-1B_, bla_CTX-M-15_, bla_VIM-1_*	*aph(3′)-XV, aacA4, aadA1, aac(3)-IId, strA, strB, mph(A), sul1, sul2, aac(6′)Ib-cr, dfrA14, dfrA17, qnrS1, tet(A), catB2*	*gyrA* S83L/D87N *parC* S80I/E84V *parE* I529L
IPABRS_VI	2	IncFII, IncFIA, Col(MG828) /F2:A1	*bla_CTX-M-15_,* *bla_TEM-1B_*		*gyrA* S83L/D87N *parC* S80I/E84V *parE* I529L
	2	IncFIA, Col (MG828), IncFIB (AP001918), Col156 /F1:A1:B10	*bla_TEM-1A_,* *bla_SHV-12_*	*mph(A), sul2, sul1, dfrA17, qnrB19, tet(A)*	*gyrA* S83L/D87N *parC* S80I *parE* L416F
IPABRT_VI	2	IncFII(AP001918), IncFII(pRSB107), Col8282, IncFIA, Col156, Col(MG828) /F1:A2:B20	*bla_CTX-M-27_*	*strB, aadA5, strA, sul1, sul2, dfrA17, tet(A)*	*gyrA* S83L/D87N *parC* S80I/E84V *parE* I529L
	2	IncFII(AP001918), IncFII(pRSB107), Col8282, IncFIA, Col156, Col(MG828) /F1:A2:B20	*bla_CTX-M-27_*	*strA, aadA5, strB, mph(A), sul1, sul2, dfrA17, tet(A)*	*gyrA* S83L/D87N *parC* S80I/E84V *parE* I529L
	1	IncFII(pRSB107), Col156, IncFIB(AP001918), IncFIB(pQil), IncFIA /IncF: F1:A2:B20	*bla_TEM-1A_, bla_OXA-1_, bla_KPC-3,_ bla_OXA-9_, bla_CTX-M-27_*	*aac(3)-IIa, aac(6′)Ib-cr, dfrA14, qnrB66, catB4*	*gyrA* S83L/D87N *parC* S80I/E84V *parE* I529L
	1	IncFIA,IncFIB(AP001918), IncFII, ColRNAI /F31:A20:B1	*bla_CTX-M-15_,bla_OXA-1_*	*catB4*	*gyrA* S83L/D87N *parC* S80I/E84V *parE* I529L
IPABSC_VI	2	IncFIB(AP001918), IncFII(pRSB107), Col8282, IncFIA, ColRNAI, Col156, IncI1 /F1:A2:B20	*bla_CTX-M-27_*	*strB, aadA5, strA, mph(A), sul1, sul2, dfrA17, tet(A)*	*gyrA* S83L/D87N *parC* S80I/E84V *parE* I529L
	1	IncFIB(AP001918), IncFII(pRSB107), IncFIA, Col156 /F1:A2:B20	*bla_CTX-M-27_*	*strB, aadA5, strA, mph(A), sul1, sul2, dfrA17, tet(A)*	*gyrA* S83L/D87N *parC* S80I/E84V *parE* I529L
POBB_VR	1	IncFIA, IncFIB(AP001918), IncFII(pRSB107), Col8282, IncN, Col156, ColRNAI /F1:A2:B20 IncN: ST7	*bla_CTX-M-15_,* *bla_TEM-1B_*	*aac(3)-IId, strA, strB, aadA5, mph(A), sul1, sul2, dfrA17, dfrA14, qnrS1, tet(A)*	gyrA S83L/D87N parC S80I/E84V parE I529L
POVI_VR	1	IncFIA, IncFIB(AP001918), IncFII, ColRNAI /F31:A20:B1	*bla_CTX-M-15_,bla_OXA-1_*	*aac(3)-IIa, aac(6′)Ib-cr, tet(A), catB4*	gyrA S83L/D87N *parC* S80I/E84V *parE* I529L
POSC_VR	2	IncFIB(AP001918), ColRNAI, IncI1, IncFII, IncFIA /F36:A20:B1	*bla_CTX-M-15_,bla_OXA-1_*	*aac(3)-IIa, aac(6′)Ib-cr, tet(A), catB4*	*gyrA* S83L/D87N *parC* S80I/E84V *parE* I529L
POCS_VR	1	IncX3, ColRNAI, IncFIB(pB171), IncI1, IncP1, FIA (pBK30683), IncFIC(FII) /F46:A6:B47	*bla_CMY-42_, bla_NDM-4_*	*tet(B)*	*gyrA* S83L/D87N *parC* S80I *parE* S458A
	1	IncFII(29), IncFIB(AP001918), ColRNAI, IncFIB(pQil) /IncF: F29:B10	*bla_CTX-M-15_,bla_OXA-9_,bla_TEM-1A_,bla_KPC-3_,* *bla_OXA-1_*	*aac(6′)-Ib-cr*	*gyrA* S83L/D87N *parC* S80I/E84V *parE* I529L

**Table 4:** Distribution of β-lactamase genes among *E. coli* strains.

*E. coli*	ST12	ST48	ST69	ST95	ST131	ST410	ST1193
Total No.	2	1	3	1	32	1	3
*bla*_TEM-1_	1	1	1	-	9	-	3
*bla*_CTX-M-1_	1	-	-	-	-	-	-
*bla*_CTX-M-3_	-	-	-	-	3	-	-
*bla*_CTX-M-15_	-	-	-	-	17	-	-
*bla*_CTX-M-27_	-	-	-	-	12	-	-
*bla*_SHV-12_	-	1	-	-	-	-	2
*bla*_KPC-3_	-	-	-	-	2	-	1
*bla*_NDM-4_	-	-	-	-	-	1	-
*bla*_VIM-1_	1	-	3	1	-	-	-
*bla*_CMY-42_	-	-	-	-	-	1	-
*bla*_OXA-1_	-	-	-	-	13	-	-
*bla*_OXA-9_	-	-	-	-	2	-	1

## Data Availability

Data sharing not applicable.
